# Division of labor and brain evolution in insect societies: Neurobiology of extreme specialization in the turtle ant *Cephalotes varians*

**DOI:** 10.1371/journal.pone.0213618

**Published:** 2019-03-27

**Authors:** Darcy Greer Gordon, Alejandra Zelaya, Ignacio Arganda-Carreras, Sara Arganda, James F. A. Traniello

**Affiliations:** 1 Department of Biology, Boston University, Boston, MA, United States of America; 2 Ikerbasque, Basque Foundation for Science, Bilbao, Spain; 3 Department of Computer Science and Artificial Intelligence, Basque Country University, San Sebastian, Spain; 4 Donostia International Physics Center (DIPC), San Sebastian, Spain; 5 Departamento de Biología y Geología, Física y Química Inorgánica, Área de Biodiversidad y Conservación, Universidad Rey Juan Carlos, Madrid, Spain; 6 Graduate Program for Neuroscience, Boston University, Boston, MA, United States of America; Universidade de Sao Paulo Faculdade de Filosofia Ciencias e Letras de Ribeirao Preto, BRAZIL

## Abstract

Strongly polyphenic social insects provide excellent models to examine the neurobiological basis of division of labor. Turtle ants, *Cephalotes varians*, have distinct minor worker, soldier, and reproductive (gyne/queen) morphologies associated with their behavioral profiles: small-bodied task-generalist minors lack the phragmotic shield-shaped heads of soldiers, which are specialized to block and guard the nest entrance. Gynes found new colonies and during early stages of colony growth overlap behaviorally with soldiers. Here we describe patterns of brain structure and synaptic organization associated with division of labor in *C*. *varians* minor workers, soldiers, and gynes. We quantified brain volumes, determined scaling relationships among brain regions, and quantified the density and size of microglomeruli, synaptic complexes in the mushroom body calyxes important to higher-order processing abilities that may underpin behavioral performance. We found that brain volume was significantly larger in gynes; minor workers and soldiers had similar brain sizes. Consistent with their larger behavioral repertoire, minors had disproportionately larger mushroom bodies than soldiers and gynes. Soldiers and gynes had larger optic lobes, which may be important for flight and navigation in gynes, but serve different functions in soldiers. Microglomeruli were larger and less dense in minor workers; soldiers and gynes did not differ. Correspondence in brain structure despite differences in soldiers and gyne behavior may reflect developmental integration, suggesting that neurobiological metrics not only advance our understanding of brain evolution in social insects, but may also help resolve questions of the origin of novel castes.

## Introduction

Adaptive morphology and neuroarchitecture support specialized behavior in complex social systems [1–5]. In the eusocial Hymenoptera, females exhibit a primary reproductive division of labor into fertile (queen) and sterile (worker) castes that potentially have identical genomes [6]. In most species, queens are morphologically and behaviorally adapted for dispersal by flight; dealate inseminated queens then transition from nest establishment and nursing behaviors during colony foundation to reproduction [7,8]. These state changes in behavior are associated with neurobiological changes in the brain [9,10]. Division of labor also concerns the differentiation of sterile workers into physical subcastes that show task specializations [11–18]. The brains of workers in different subcastes as well as those of queens and workers may be distinguished by the relative proportion of neuropil in functionally specialized brain regions that correlate with morphological and behavioral differentiation [19–23].

The behavioral demands of colony foundation and reproduction have neurobiological correlates [9,10,20,21] and brain structure has been demonstrated to be associated with subcaste division of labor in some ants [22,24–26]. In taxa with distinct castes and worker subcastes, size, form, and behavioral specialization are predicted to be integrated with neuroarchitecture, but these relationships, and their developmental linkages, are not well understood. Indeed, worker polymorphism, age, and the development and differentiation of social roles can be reflected in divergent neuroanatomical phenotypes in some polymorphic ant species [5,22], but conserved in others [25]. At the cellular level, behavioral differentiation of female phenotypes may also be associated with the structure of synaptic complexes termed microglomeruli (MG) formed from sensory neuron projection boutons and dendritic spines of mushroom body (MB) intrinsic Kenyon cells [27,28]. The organization of these microglomeruli, which may enable neural plasticity and support behavioral diversity, has been used as a proxy for information-processing abilities that appear to correlate with task performance demands [28–32]. However, MG structure may not be consistently associated with worker size-related behavioral differentiation [25,31–33].

**Fig 1:**
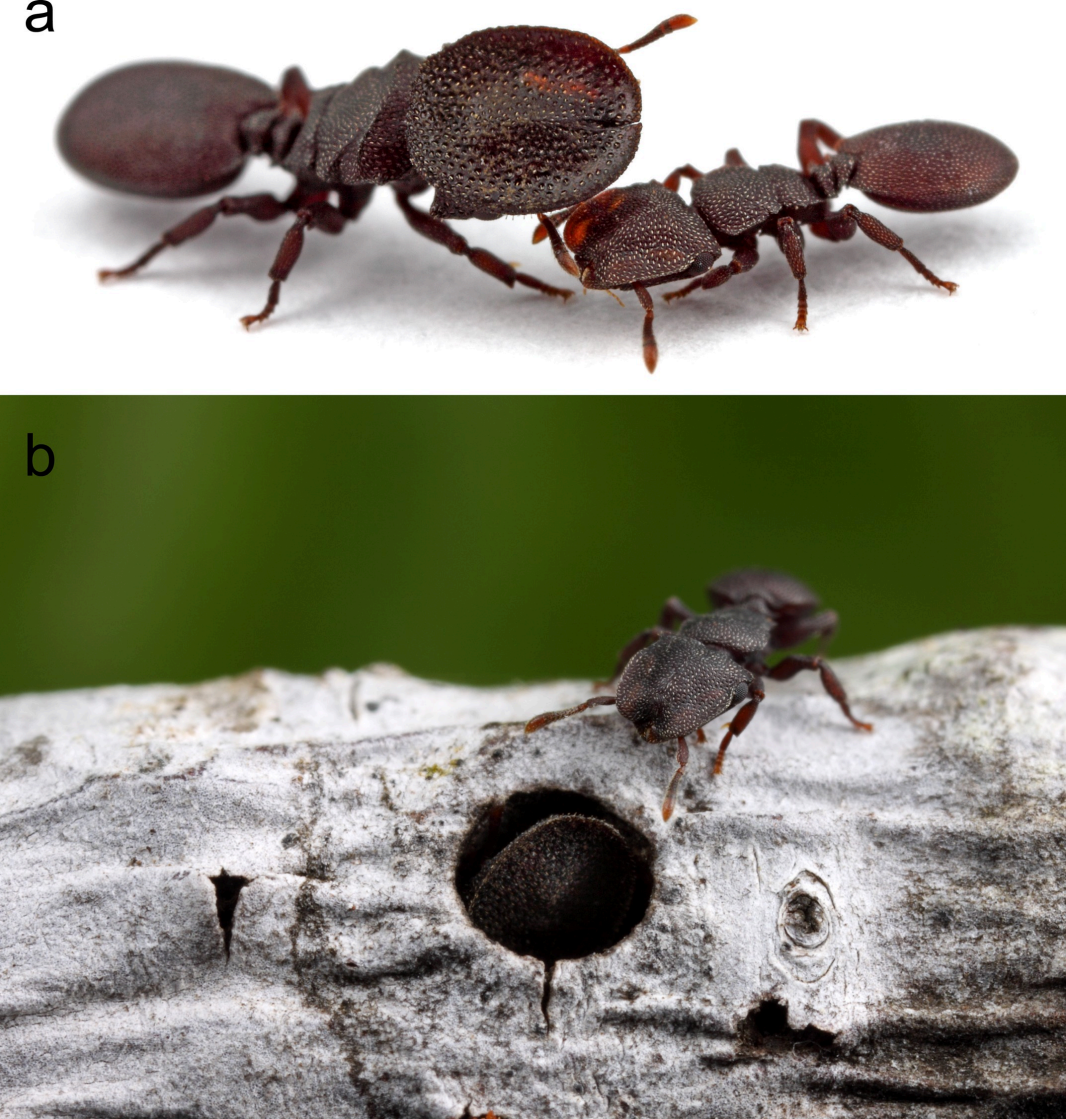
Extreme morphological differentiation in *C*. *varians*. Soldier (A, left) compared to a minor workers (A, right). (B) Soldier using shield-shaped head to block nest entrance (minor worker above). Photo credit: Alexander Wild.

Ant species characterized by exceptional patterns of caste and subcaste evolution provide important models to examine relationships of morphology, brain structure, and behavioral specialization in association with division of labor and related elements of social complexity [5,22,32,34]. Social organization in the turtle ant *Cephalotes varians* is characterized by a reproductive (queen) caste and dimorphic subcastes of small minor workers and larger highly specialized phragmotic soldiers that use their shield-shaped heads as physical barriers to control entry to the nest (Fig 1). Soldiers, which serve as “living doors,” perform few behaviors apart from their important role in colony security [35,36]. *C*. *varians* gynes, like soldiers, have shield-shaped heads used to block nest entrances during haplometrotic (single queen) colony foundation [S. Powell, personal communication] but later in colony development become specialized on reproduction. The extraordinary polymorphism associated with division of labor in *C*. *varians* thus involves striking variation in size and extreme differences in morphology and social function. In light of this caste and subcaste differentiation, we hypothesized that brain size, compartment scaling, and synaptic organization would correlate with reproductive and worker roles, particularly the remarkable morphology of soldiers and their specialized behavior. Due to similarity in body size and head morphology, we predicted soldiers and gynes would have significantly larger brains than minor workers, and that brain structure in mature *C*. *varians* minor workers would be characterized by large MBs, centers of learning, memory, and sensory integration, due to their significantly larger behavioral repertoire [35,36] in comparison to soldiers and gynes. We hypothesized that brains of soldiers and gynes, which share some morphological characteristics likely due to common developmental pathways, would be less differentiated, and that patterns of synaptic organization would be similar across female phenotypes due to the integration of brain compartment scaling and MB synaptic circuitry. Based on prior studies of task specialization in polymorphic ants [31–33], we expected minor workers would have smaller and more dense MG compared to soldiers and gynes, reflecting higher demands for processing social information and environmental cues, and potentially, developmental constraints.

## Methods

### Ant collection and culture

Queenright *C*. *varians* colonies and colony fragments containing minor workers, soldiers, and winged gynes were collected in red mangrove stands in March, 2015 in the Florida Keys: Key West (24.55793° N; 081.76278° W), No Name Key (24.69786° N; 081.34054° W), and Key Largo (25.12404° N; 080.40276° W). Collection permits were issued by the U.S. Fish and Wildlife Service (# FFO4RFKD-2015-0) and the Florida Department of Environmental Protection (# 0127201515). Collections did not involve endangered or protected species. Colonies were cultured in an environmental chamber at 25°C, 65% humidity with a 12h:12h light:dark cycle in test tubes partially filled with water and plugged with cotton placed inside small (17cm x 12cm) plastic boxes coated with Fluon (BioQuip) with ventilated lids. Colonies were provisioned with 1M sucrose every other day and pine pollen *ad libitum*. Head width at the widest point (HW) was measured for all individuals sampled, average HW ± standard deviation are reported. We used female phenotype as a categorical variable in all analyses because HW, brain volume, and body size appear to be decoupled in *C*. *varians*.

### Brain size and compartment scaling

Brains of minor workers (HW = 1.15mm ± 0.075, N = 20), soldiers (HW = 1.63 ± 0.075, N = 20), and gynes (HW = 1.61 ± 0.070, N = 20) sampled from four queenright colonies and seven colony fragments were processed using a modified immunohistochemical protocol [37,38]. Brains were dissected in ice cold HEPES-buffered saline (HBS) and fixed overnight at 4°C on a shaker in 1% zinc formaldehyde. A series of HBS washes (6 x 10 minutes) was followed by further fixation in Dent’s fixative (4:1 methanol: dimethyl sulfoxide) for 1–2 hours before brains were stored in 100% methanol. When ready for further processing, brains were rehydrated in 0.1 M Tris buffer and blocked for one hour in a normal goat serum (NGS) solution (PBSTN: 5% NGS + 0.005% sodium azide in 0.2% Triton-X phosphate buffered saline [PBST]). Brains were then incubated for four nights at 4°C on a shaker in primary antibody, SYNORF1 (AB_2315426; Developmental Studies Hybridoma Bank), diluted 1:30 in PBSTN. Brains were washed again (6 x 10 minutes in 0.2% PBST) and incubated for another three nights wrapped in foil at 4°C on a shaker in AlexaFluor488 (ThermoFisher) goat anti-mouse secondary antibody (1:100 in PBSTN). Brains were washed again (6 x 10 minutes in 0.2% PBST) and dehydrated in a series of ethanol:PBS solutions (5 minutes each in 30%, 50%, 70%, 95%, 100%, 100%) and stored overnight at -20°C wrapped in foil. Finally, brains were cleared and mounted with methyl salicylate in stainless steel well slides. An Olympus Fluoview BX50 laser scanning confocal microscope was used to image cleared brains with either a 10X (NA = 0.3) or 20X (NA = 0.5) objective. To produce image stacks of slices with a true thickness of ~5 μm, optical sections in the horizontal plane (3.1 μm steps) were captured and corrected along the z-axis (by a factor 1.59) because of axial distortion produced by the refractive index mismatch between air and methyl salicylate [22]. To confirm that the same correction factor could be used for the two objectives despite their different numerical aperture, we imaged brains at both 10X and 20X and found that the objectives produced indistinguishable axial distortion.

### Construction of brain templates

The sizes of functionally distinct brain regions—the optic lobe excluding the lamina (OL, visual input), antennal lobe (AL, olfactory input), mushroom body calyces (MBC, integrative sensory input), mushroom body peduncle and lobes (MBP, integrative sensory output and modulation), central complex (CX, motor integration and spatial navigation), and subesophageal zone (SEZ, gustation and head movement), together with the rest of the undifferentiated central brain (ROCX)—were quantified for one hemisphere of each brain. Compartment volumes were obtained for each individual by automatic labeling, using brain templates for minor workers, soldiers, and gynes created in diffeomorphic space [39,40]. Diffeomorphisms are smooth invertible mappings of 3-D images commonly used in computational neuroimaging; their topological properties enable the co-registration of brains to measure structural variation [39,41]. *C*. *varians* brain templates consisted of 3-D average-shape brains resulting from the combination of N co-registered original brains (N = 10) from confocal scans. Difference in illumination of the original brains were first corrected using histogram matching [42]. Co-registration was then based on an initial affine transformation that maximizes mutual information between brain volumes, thus identifying common neuropil shape, followed by an iterative refinement based on local non-rigid transformations that maximize the cross-correlation of voxel intensities of co-registered brains. Final average shapes were created using a voxel-wise median over co-registered brains. In a modification of the protocol described in Arganda-Carreras et al. [39], we did not build consensus labels, but manually traced brain regions for each template using Amira (FEI v 6.2.0). Brain regions that span the midline (CX and SEZ) were traced in their entirety and later divided in half to give volumes equivalent to one hemisphere. The axis of symmetry was used to demarcate boundaries for the ROCX. All other neuropil (OL, AL, MBC, and MBP) have clearly delineated boundaries. To automatically trace brain regions, we registered individual brains against templates and for each brain applied the reverse transform on the manual labels of a template to automatically label brain regions. All automatic labels were validated in Amira (FEI v 6.2.0) to reduce the chance of inaccuracies, and if needed, manual corrections were made before calculating regional volumes. All procedures except the manual tracing of brain regions were implemented using Advanced Normalization Tools (ANTs) software [43].

### Statistical analysis of brain structure

We used analysis of variance (ANOVA) to test for the effect of female phenotype on the volume of the whole brain hemisphere as a proxy metric of total brain size, and post-hoc Tukey Honest Significant Difference (HSD) tests to determine pairwise differences between female groups. Scaling relationships (slope, shift, elevation) of brain region volumes with the rest of the hemisphere volume (RH = OL + AL + MBC + MBP + CX + SEZ + ROCX–region of interest) were determined using standard major axis regression analysis (SMA) to compare female phenotypes with the package smatr [44] in R (version 3.3.2). If brain regions of female phenotypes had similar slopes (shared β), 95% confidence intervals (CI) were calculated for the slope, and Pearson’s chi-squared (*χ*^*2*^) was used to determine if the common slope significantly differed from one. Differences among shift (axis shift) and elevation (grade shift) were examined using the Wald statistic (*W*^*2*^). Significant axis shifts indicate differences across groups in mean size, whereas grade shifts indicate differences in relative investment in a particular brain compartment at a similar x value.

Hierarchical cluster and discriminate function analyses were employed to examine the degree of separation of brain phenotypes in multivariate space. To control for variation in overall brain size, the proportion of neuropil in each functional subregions (OL, AL, MBC, MBP, CX, SEZ) was calculated by dividing the region of interest volume by the volume of the whole hemisphere. Hierarchical cluster analysis using Euclidean distance and average linkage was used to assess groupings according to relative neuropil size without *a priori* assignment by caste or subcaste. Multiscale bootstrapping methods (N = 10,000) were used to assign approximately unbiased (AU) *p*-values using the package pvclust to determine if clusters had significant support [45]. We used discriminate function analyses to determine how accurately individual brains could be classified into *a priori* groups. This was visualized by presenting inertia ellipses overlaid on the biplot of the discriminant factors per female phenotype via package ade4 [46]. Press’s Q statistic was calculated for both the full model and a leave-one-out cross-validation model to assess whether groupings were classified at a probability greater than chance.

### Microglomeruli (synaptic) structure

Brains from minor workers (HW = 1.08 ± 0.073, N = 14), soldiers (HW = 1.54 ± 0.052, N = 10) and winged gynes (HW = 1.54 ± 0.034, N = 11) sampled from three queenright lab colonies were processed according to modified protocols [30,47]. Brains were dissected in ice-cold HEPES-buffered saline and fixed at 4°C overnight on a rotator in 4% paraformaldehyde in 0.1 M PBS. Brains were washed (3 x 20 minutes) in 0.1 M PBS, before embedding in low melting point agarose (5.5 g/mL PBS). A Leica VT1200S vibratome was used to produce 100 μm thick sections in the horizontal plane. Sections were treated with 2% and 0.2% PBST each for 20 minutes to permeabilize the tissue before blocking for one hour at room temperature on a plate shaker in NGS (2% in 0.2% PBST). Sections were then incubated for at least three nights at room temperature while covered in foil on a plate shaker in SYNORF1 (1:50) and AlexaFluor488-phalloidin (1:500) in 0.2% PBST. Brain sections were then washed in 0.1 M PBS (5 x 20 minutes) and incubated overnight at room temperature while covered in foil on a plate shaker in AlexaFluor568 goat anti-mouse secondary antibody (1:250) in 1% NGS-PBS. Lastly, sections were incubated overnight in 60% glycerol-PBS followed by 80% glycerol-PBS (30 minutes) and then mounted in 80% glycerol-PBS on glass slides sealed with nail polish. Prepared slides were imaged without digital zoom at 1024 x 1024 pixel resolution on an Olympus Fluoview FV10i (NA = 1.4) inverted laser scanning confocal microscope with a 60X oil immersion lens. Snapshot images (1.55 μm thick) in a plane which the MB peduncle bisects the MB calyxes were captured.

One lateral and one medial calyx, coded to blind the observer to female phenotype during image processing, were imaged for each individual. Modified protocols [30–32,47,48] were used to measure MG density and size. Two adjacent circles (400 μm^2^ each) were overlaid in the non-dense lip region and one circle (400 μm^2^) was overlaid on the collar region of each calyx. All MG, excluding those intersecting the circumference lines of the circles, were counted using Fiji software at high digital magnification (300X). To measure the longest aspect of MG boutons, a grid (10 μm^2^ squares) was overlaid on the predefined circles and a random number generator was used to select five squares in which all MG within the square or intersecting the top and right borders were measured. Average density of the lip and collar MG (MG/μm^3^) were calculated for each individual from counts of MG and divided by the volume of the region. Similarly, average bouton length was calculated for each individual for the lip and collar region.

### Statistical analysis of MG structure

ANOVA was used to examine effects of female phenotype on MG density and size in the lip and collar regions. Shapiro and Bartlett tests were used to assess the assumptions of residual normality and homoscedasticity, and assumptions were met for all four MG metrics. Post-hoc Tukey HSD tests were employed to determine the significance of pairwise comparisons if the overall effect of female phenotype was determined to be significant. All analyses were conducted in R (version 3.3.2).

## Results

### Brain size and compartment scaling

**Fig 2:**
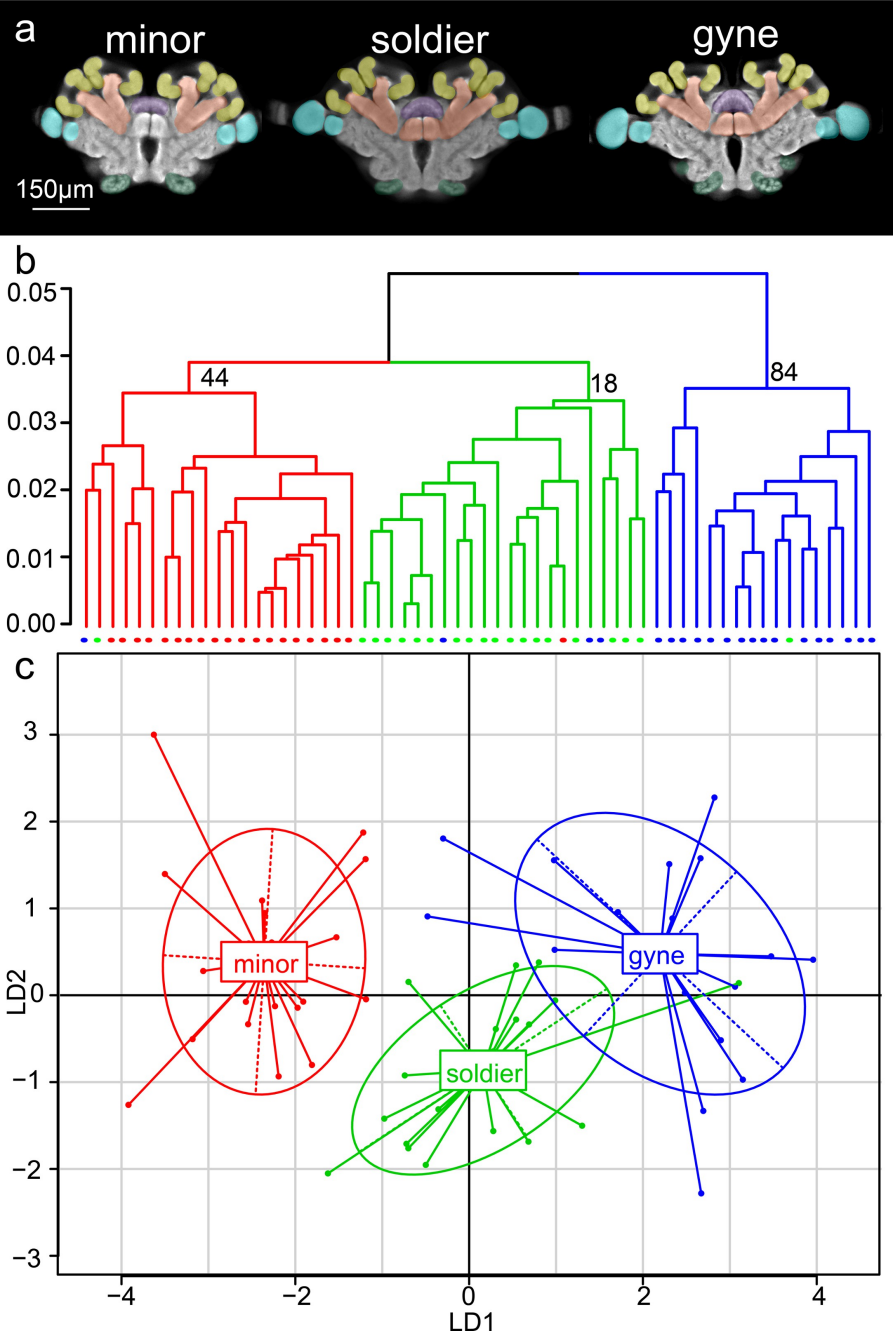
Multivariate analyses of caste and subcaste differences in brain structure. (A) False-colored template brains of gynes, soldiers, and minor workers (OL: blue, AL: green, MBC: yellow, MBP: orange, CX: purple, ROCX: gray, SEZ: not shown). (B) Dendrogram with AU p-values (%) at nodes from hierarchical analysis. (C) Inertia ellipses with center of mass determined from discriminate function analysis. (B-C) Minor: red, soldier: green, gyne: blue.

There was a significant effect of female phenotype on hemisphere volume (ANOVA: *F*_2, 57_ = 5.29, *p =* 0.008; Fig 2A). Post-hoc Tukey tests revealed that gynes had significantly larger brain volumes than minors (*p* = 0.01) and soldiers (*p* = 0.02), which did not differ from each other (*p* = 0.98). All six brain subregions had similar slopes (*p* > 0.05) across castes and subcastes; therefore, grade shifts and axis shifts were examined to explore differences in compartment proportional investment and overall mean size, respectively (Table 1 and S1 Fig). All subregions with the exception of the positively allometric AL and MBP displayed isometry with the RH. There were overall size differences indicated by axis shifts in all but the MBC and MBP. Grade shifts were evident in all regions except the AL and SEZ; gynes and majors had disproportionately large OL and CX, but minors had larger MBC and MBP.

**Table 1:** SMA analyses of log-transformed regions of interest and RH volumes for different female groups.

	OL	AL	MBC	MBP	CX	SEZ
**Shared β**	1.25	1.20	1.11	1.28	1.06	1.07
**95% CI**	1.03, 1.52	1.07, 1.34	0.98, 1.25	1.25, 1.43	0.89, 1.25	0.94, 1.22
**β ≠ 1?**	no	yes	no	yes	no	no
***χ*** ^***2***^	5.15	9.95	4.23	18.22	4.98	2.45
***p* value**	0.16	0.02	0.24	0.0003	0.17	0.48
**Grade shift**	G = S > M	no	M > S > G	M > S > G	G = S > M	no
***W***^***2***^	113.2	4.66	66.71	93.46	19.45	1.86
***p* value**	< 0.0001	0.10	< 0.0001	< 0.0001	<0.0001	0.39
**Axis shift**	G >S > M	G > S = M	no	no	G > M; S intermediate	G > M; S intermediate
***W***^***2***^	51.96	9.82	3.83	3.79	17.51	9.56
***p* value**	< 0.0001	0.007	0.15	0.15	0.0002	0.008

Abbreviations of brain regions given in Methods, G (gynes); S (soldiers); M (minors).

Although groups appeared to separate according to neural phenotype, clusters were not statistically supported by multiscale bootstrapping (gyne cluster: AU *p*-value = 0.84, SE = 0.01, soldier cluster: AU *p*-value = 0.18, SE = 0.05 minor worker cluster: AU *p*-value = 0.44, SE = 0.07; Fig 2B). However, when assigned group membership *a priori* in discriminate function analyses, individual brains were correctly classified to caste and subcaste in at least 80% of cases, a result that was significant (full model: Press’s Q = 102.68, *p* < 0.0001; leave-one-out: Press’s Q = 67.5, *p* < 0.0001; Fig 2C; see discussion).

### Synaptic structure

**Fig 3:**
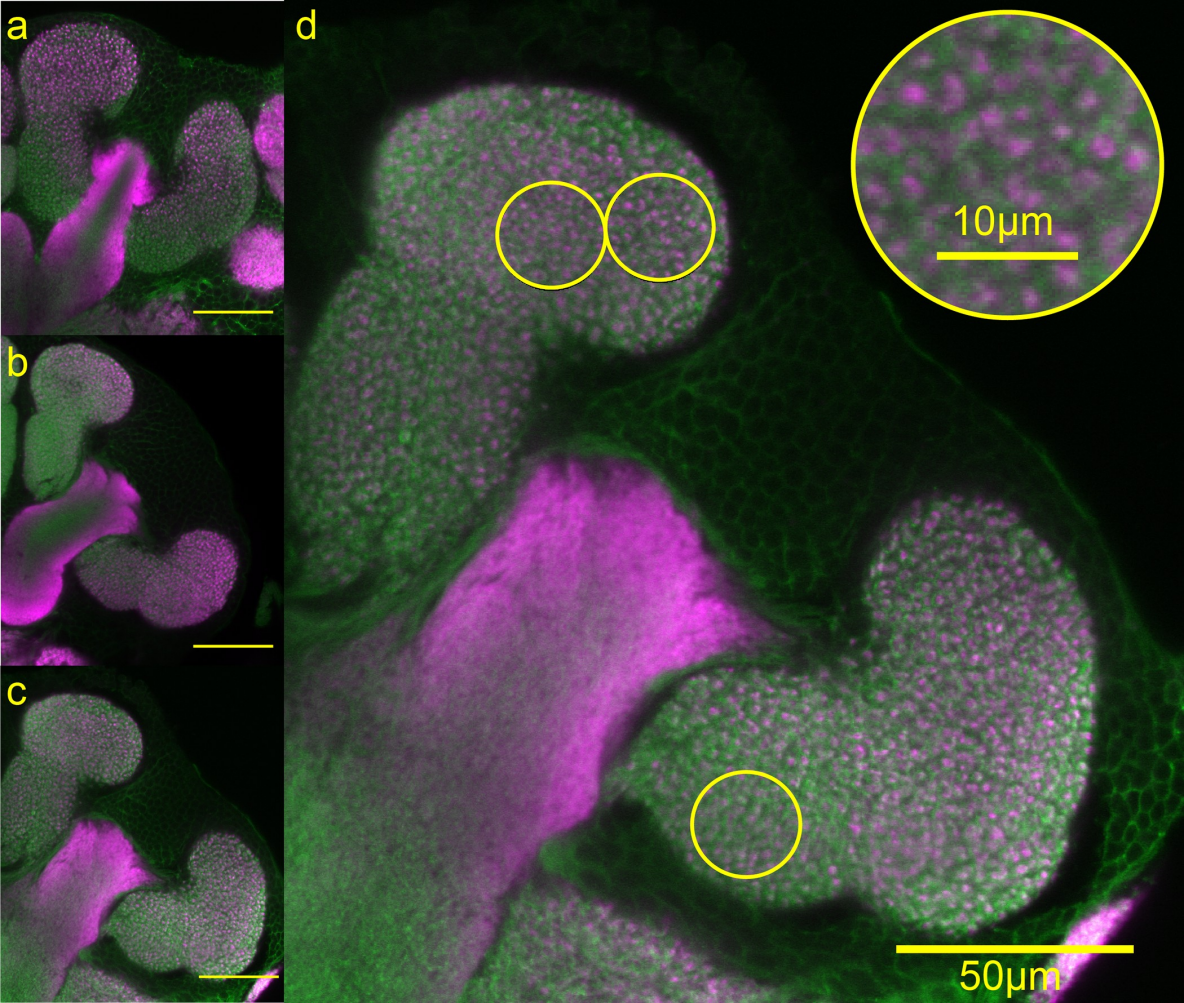
Representative micrographs of MG structure. Minor (A), soldier (B), and gyne (C) brains shown with scale bars = 50μm (A-C). MG quantification method in an image of a gyne brain (D). Inset magnification shows distal lip circle.

**Fig 4:**
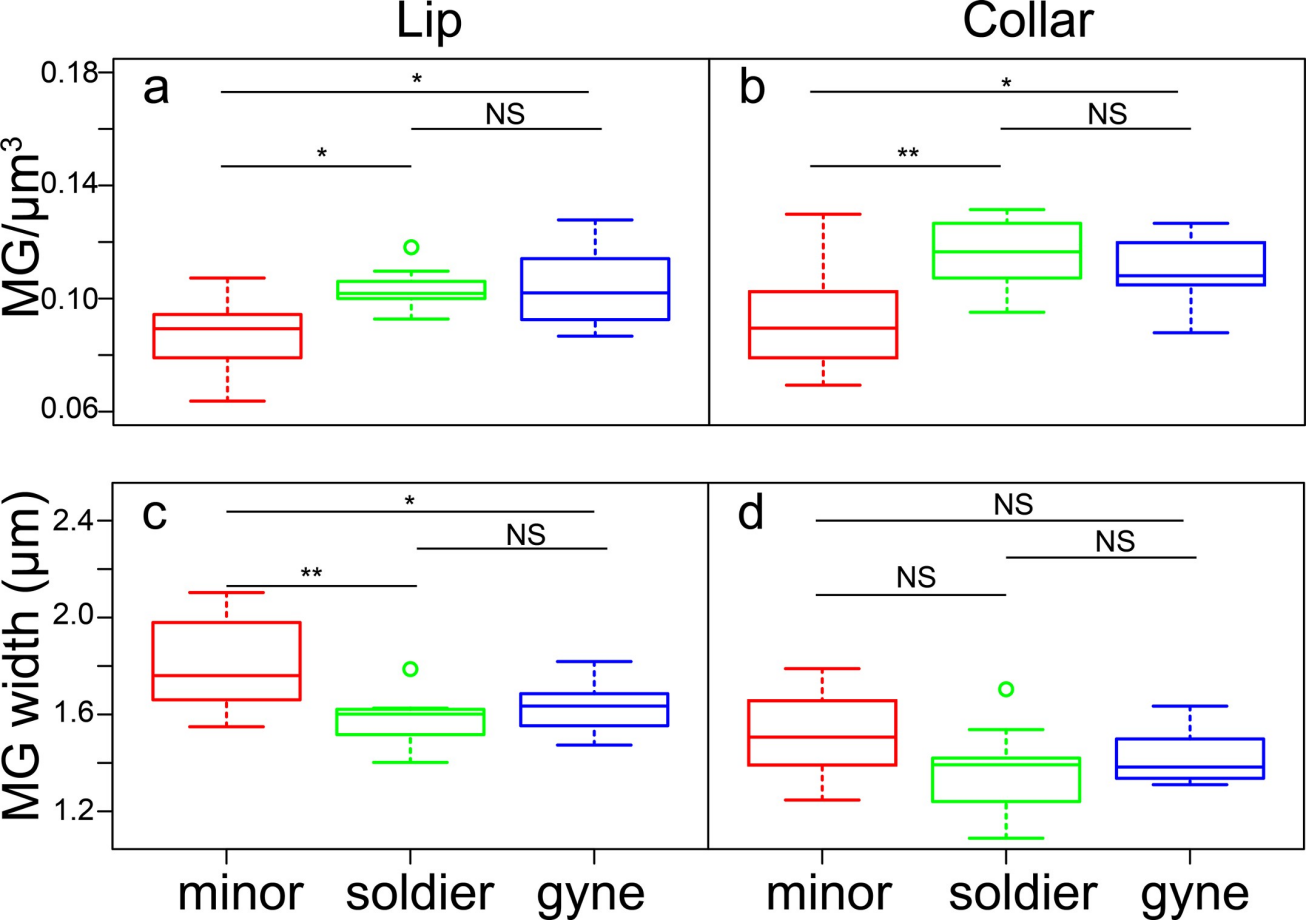
MG density and size varies across *C*. *varians* castes. MG density (A, B) and size (C, D) in the lip (A, C) and collar (B, D) in minors (N = 14), soldiers (N = 10), and gynes (N = 11). Tukey HSD *p*-value: NS > 0.05; * <0.01; ** <0.001.

Minor workers, soldiers, and gynes varied in the density and size of MG (Fig 3). Female phenotype significantly affected MG density in the lip (ANOVA: *F*_2, 32_ = 8.94, *p <* 0.001; Fig 4A) and collar regions of the MB (ANOVA: *F*_2, 32_ = 9.84, *p <* 0.001; Fig 4B). Minor workers had significantly less dense MG than soldiers and gynes. Caste and subcaste identity significantly affected average bouton size in the lip (ANOVA: *F*_2, 32_ = 9.21, *p <* 0.001; Fig 4C); minor workers had larger boutons. Similarly, minor workers had larger boutons in the collar, but the difference was not significant (ANOVA: *F*_2, 32_ = 2.62, *p* = 0.088; Fig 4D). In all comparisons, soldiers and gynes did not significantly differ in MG metrics (Tukey HSD tests *p* > 0.05).

## Discussion

Contrary to our body-size based prediction, *C*. *varians* gyne brains were significantly larger than those of soldiers and minors, which did not differ. However, our predictions regarding brain substructure scaling across castes and subcastes were supported: minors invested proportionally more in MBs and gynes had absolutely and relatively larger OLs and CXs. Soldier brain structure appeared to be intermediate between that of minors and gynes. Although the three groups separated neuroanatomically in multivariate space, significant differences were found only when group identity was specified *a priori* in discriminate analysis, indicating greater overlap in brain structure between castes and subcastes, but differences in relative volumes of the OLs and MBs.

### Functional significance of *C*. *varians* brain compartment scaling and synaptic structure

*Cephalotes varians* minor workers had proportionally larger MBs and smaller OLs than gynes. Increased relative size of the OL is associated with the use of vision during flight in paper wasp workers [21], and *C*. *varians* gynes would appear to overlap in the demands for processing visual information during dispersal by flight from the parent nest and thus show neurobiological convergence. However, allometrically large MBs in paper wasps may involve cognitive demands associated with reproductive competition [20], whereas the diverse task repertoire of sterile *C*. *varians* minor workers may require greater processing capabilities and larger MB size. Whether the relatively small OLs of minor workers represent adaptive size variation due to less demanding processing of visual information or developmental constraint due to their smaller body size is unclear. Soldiers have the largest body size of army ant (*Eciton*) workers, but brain size (specifically MBs and ALs) in this subcaste is allometrically small [26], further supporting an association of brain investment pattern and social function. However, brain structure in army ant reproductives has not been studied.

Comparisons of MG structural variation across *C*. *varians* female phenotypes support the hypothesis that extreme behavioral specialization is reflected in synaptic organization, although not consistently in the manner we predicted. MG metrics were similar in soldiers and gynes, suggesting morphology and this aspect of cellular brain organization are coupled, in spite of apparent caste and subcaste life-history differences in behavior. Gynes fly from their parental nest, mate, shed their wings, and then initiate new colonies. Flight likely requires navigational abilities, and colony foundation involves performing diverse tasks ranging from nursing immatures to blocking intruders from entering nests. Only nest-entrance guarding by gynes appears to overlap with the soldier repertoire, although soldiers could function in food storage [12]. Nevertheless, MG organization in gynes and soldiers is similar, suggesting they share genetic architectures that guide brain development.

Microglomeruli were larger and less dense in minor workers than in soldiers and gynes; this pattern of MG density and size across female phenotypes was contrary to our prediction that minor workers would have more densely packed MG, underpinning the need for cognitive processing associated with their pluripotent task repertoire. We can interpret the significance of this result by evaluating findings of prior studies that describe diverse correlates of MG structure in eusocial hymenopterans. For example, MG density, size, and number have been associated with temperature during development [49–51], learning and memory [52,53], behavioral maturation [48,54–59], resilience to aging [47], robustness to sensory injury [60], and division of labor [25,31,33]. Higher MG density correlates interspecifically with greater social complexity [25,30], supporting a role for MG density in processing social information. Intraspecific comparisons of patterns of MG structure between reproductives and workers or across polymorphic workers, however, are inconsistently associated with division of labor. Honeybee queens, which specialize on egg-laying, have larger and less dense MG in smaller MBCs than workers, perhaps contributing to their relatively weak learning performance [51,61]. In the strongly polymorphic fungus-growing ant *Atta vollenweideri*, MG density is constant across size-variable workers; larger workers have correspondingly larger brains and absolutely more MG than smaller workers [33]. Although fewer MG could constrain cognitive processing abilities, reduced task repertoires may not reflect MG number. Kamhi et al., for example, found no significant differences in MG between task-generalist weaver ant (*Oecophylla smaragdina*) major workers and apparently behaviorally limited brood-care specialist minor workers when interactions with age were excluded [25]. Increases in MG density may be transient, occurring in response to learning [52,53], and decreases in MG density may be associated with age, visual experience, and development of navigational ability [48,54]. However, in some ant species MG density in the collar [59] or lip [52] increases for several weeks post eclosion. Comparisons between mature (20 day) and 95 day (old) *Pheidole dentata* minor workers show no significant differences in lip MG density or behavioral performance, suggesting MG structure in this ant resists aging [47].

The temporal dynamics of MG organization suggest that variation in individual experience and corresponding variation in MG structure is superimposed on a robust plastic neural architecture influenced by age and associated with behavioral maturation [27]. Although we did not examine age effects on brain structure in our present study, the concept of repertoire expansion, the age-related increase in task diversity [62] and efficiency [63], may provide insight into how higher-order processing demands relate to synaptic organization in *C*. *varians*. Electron microscopy (EM) of *P*. *dentata* minor worker brains showed decreases in lip bouton density and increases in bouton size and number of synapses and vesicles with increasing minor worker age [64]. Quantifying MG immunohistochemically, as we did here, provides a more rapid but less detailed assessment of synaptic remodeling that yields similar results to EM [55]. Increased behavioral capability is generally associated with decreased MG density and increased bouton size [48,54–57], and increased relative volume of MBs [22,65].

Consistent with these patterns, *C*. *varians* task-generalist minor workers had larger, less dense MG in their disproportionately large MBCs compared to more specialized soldiers and gynes. However, *C*. *varians* gynes have a more extensive repertoire than soldiers but similar MG structure, although minors show more diverse behavior. This may be because we assessed virgin gyne brain structure, which likely changes after mating and during colony foundation [9]. MG changes may occur during the incipient stages of colony growth, when queens perform a wide array of tasks. Differences in MG density and size may thus be a function of experience-expectant or experience-dependent development. Soldiers, gynes, and inexperienced (newly eclosed) minors could have small and more dense MG, but with experience, synaptic pruning and bouton expansion may occur, reflected in MG structure. Since mature soldiers are extremely specialized and apparently limited in experience, they may not undergo synaptic remodeling. However, after leaving the natal nest or following insemination, neuronal activity that may trigger synaptic remodeling [66] could occur in the brain of gynes.

### Comparative analysis of brain structure in extremely polymorphic sister clades

Our prior analysis of the integration of morphology, brain structure, and behavior in workers of *Pheidole rhea*, a sister clade also characterized by extreme variation in worker form, found significant differences in brain structure and morphology between minor workers and the two larger size classes of soldiers [5], although behavioral repertories broadly overlapped among worker size classes. Moreover, we identified only minor differences in social information processing (trail-pheromone responsiveness) across *P*. *rhea* size-variable workers [34]. In this species, MG density mainly varied between the smallest (minors) and largest (supersoldiers) worker size classes, and soldiers, which are intermediate in size did not significantly differ from the other two worker size classes in most measures of MG structure [32]. MG density and size did not consistently correlate with behavioral repertoire size and relative MB size in *P*. *rhea* workers, thus a robust relationship between synaptic structure and repertoire size could not be identified. In *C*. *varians*, minor worker and soldier brains did not differ in size, but MBs were disproportionally larger in minors in association with their more pluripotent behavior, and MG structure in *C*. *varians* minors, soldiers, and gynes indicated that the remarkable task specialization in this species broadly correlates with synaptic organization, but not always as anticipated.

### Brain structure and caste evolution

Analyses of brain structure may provide insight into caste origins given that neural phenotypes evolutionarily link morphology and behavior [13,26,67–72]. Soldiers are thought to have originated by modifying the developmental trajectory of queens [67], reprogramming worker larval growth rules [73], combining queen and worker developmental modules [68–70], or altering size-related caste determination mechanisms [72]. Recently, soldier development in *Pheidole* was found to be regulated by rudimentary wing discs [74]. Our macroscopic and synaptic study of caste- and subcaste-related brain structure in *C*. *varians* found that soldier brains are intermediate between minors and gynes. Soldier brain volumes do not significantly differ from those of minors, but are significantly smaller than those of queens, suggesting that brain size is developmentally uncoupled from body size. Although brain size does not differ between minor and soldiers, regions vary in relative volume and average size, and soldier/gyne similarity is reflected in MG structure and OL. Patterns of soldier brain size and compartment scaling are consistent with a mosaic model of brain evolution. Although this could suggest an intercaste origin of soldiers, brain gene expression studies across castes and subcastes are needed to differentiate between alternative hypotheses of novel caste origins and determine how novel castes co-opt developmental modules retained in the female genome.

## Supporting information

S1 Fig Scaling relationships of log-transformed volumes of regions of interest against log-transformed RH. Gynes (blue), soldiers (green), minors (red). Abbreviations of brain regions of interest given in Methods.
(TIF)
