# Correction: Division of labor and brain evolution in insect societies: Neurobiology of extreme specialization in the turtle ant *Cephalotes varians*

**DOI:** 10.1371/journal.pone.0219036

**Published:** 2019-06-24

**Authors:** Darcy Greer Gordon, Alejandra Zelaya, Ignacio Arganda-Carreras, Sara Arganda, James F. A. Traniello

In the Author Contributions, James F. A. Traniello (JFAT) should be listed as a contributor to Conceptualization and Project Administration.
